# Millennial climate oscillations controlled the structure and evolution of Termination II

**DOI:** 10.1038/s41598-020-72121-4

**Published:** 2020-09-10

**Authors:** David Domínguez-Villar, Juan A. Vázquez-Navarro, Kristina Krklec, Sonja Lojen, José A. López-Sáez, Miriam Dorado-Valiño, Ian J. Fairchild

**Affiliations:** 1 Department of Soil Science, Faculty of Agriculture, University of Zagreb, Svetošimunska 25, 10000 Zagreb, Croatia; 2 School of Earth and Environmental Sciences, University of Birmingham, Edgbaston, Birmingham, B15 2TT UK; 3 Department of Geography, Madrid Autonomous University, Ctra. de Colmenar km 15, 28049 Madrid, Spain; 4 Jožef Stefan Institute, Jamova cesta 39, 1000 Ljubljana, Slovenia; 5 School of Environmental Sciences, University of Nova Gorica, Vipavska 13, 5000 Nova Gorica, Slovenia; 6 Institute of History-Centre for Human and Social Sciences, Spanish National Research Council (CSIC), C/Albasanz 26-28, 28037 Madrid, Spain

**Keywords:** Biogeochemistry, Climate sciences, Ocean sciences

## Abstract

The controls that affect the structure and timing of terminations are still poorly understood. We studied a tufa deposit from the Iberian Peninsula that covers Termination II (T-II) and whose chronology was synchronized to speleothem records. We used the same chronology to synchronize ocean sediments from the North Atlantic to correlate major climate events in a common timescale. We identify two stages within T-II. The first stage started with the increase of boreal summer integrated solar insolation, and during this stage three millennial climate oscillations were recorded. These oscillations resulted from complex ocean–atmosphere interactions in the Nordic seas, caused by the progressive decay of Northern Hemisphere ice-sheets. The second stage commenced after a glacial outburst that caused the collapse of the Thermohaline Circulation, a massive Heinrich event, and the onset of the Bipolar Seesaw Mechanism (BSM) that eventually permitted the completion of T-II. The pace of the millennial oscillations during the first stage of T-II controlled the onset of the second stage, when the termination became a non-reversible and global phenomenon that accelerated the deglaciation. During the last the two terminations, the BSM was triggered by different detailed climate interactions, which suggests the occurrence of different modes of terminations.

## Introduction

Glacial terminations commence when ice-sheets from the Northern Hemisphere reach a supercritical size and their ablation is enhanced due to the increase of insolation during the boreal summer^1–3^. Terminations reach a point of no return when the Thermohaline Circulation shuts down abruptly and triggers the BSM that causes non-linear modifications of the global climate^4–6^. Because of progressive variations of the orbital forcing^7^, suitable temporal windows for the occurrence of the BSM exceed 10 ka. This ample time frame represents a limitation to understand the precise inception time and causes of the BSM that triggers the most significant climate changes during terminations^2,8,9^. We studied a tufa deposit from the Iberian Peninsula that clearly records millennial climate oscillations during T-II, also identified in many records of the North Atlantic and Mediterranean regions. The aim of this study is to describe a sequence of events and the mechanisms that controlled the structure and timing of the complete T-II by integrating the evidence from the Nordic Seas and the North Atlantic, along with their influence in the Mediterranean region^8,10,11^, with the response of the Southern Hemisphere once the BSM was activated^5,12^.

## Results and discussion

### Trabaque tufa record

**Figure 1:**
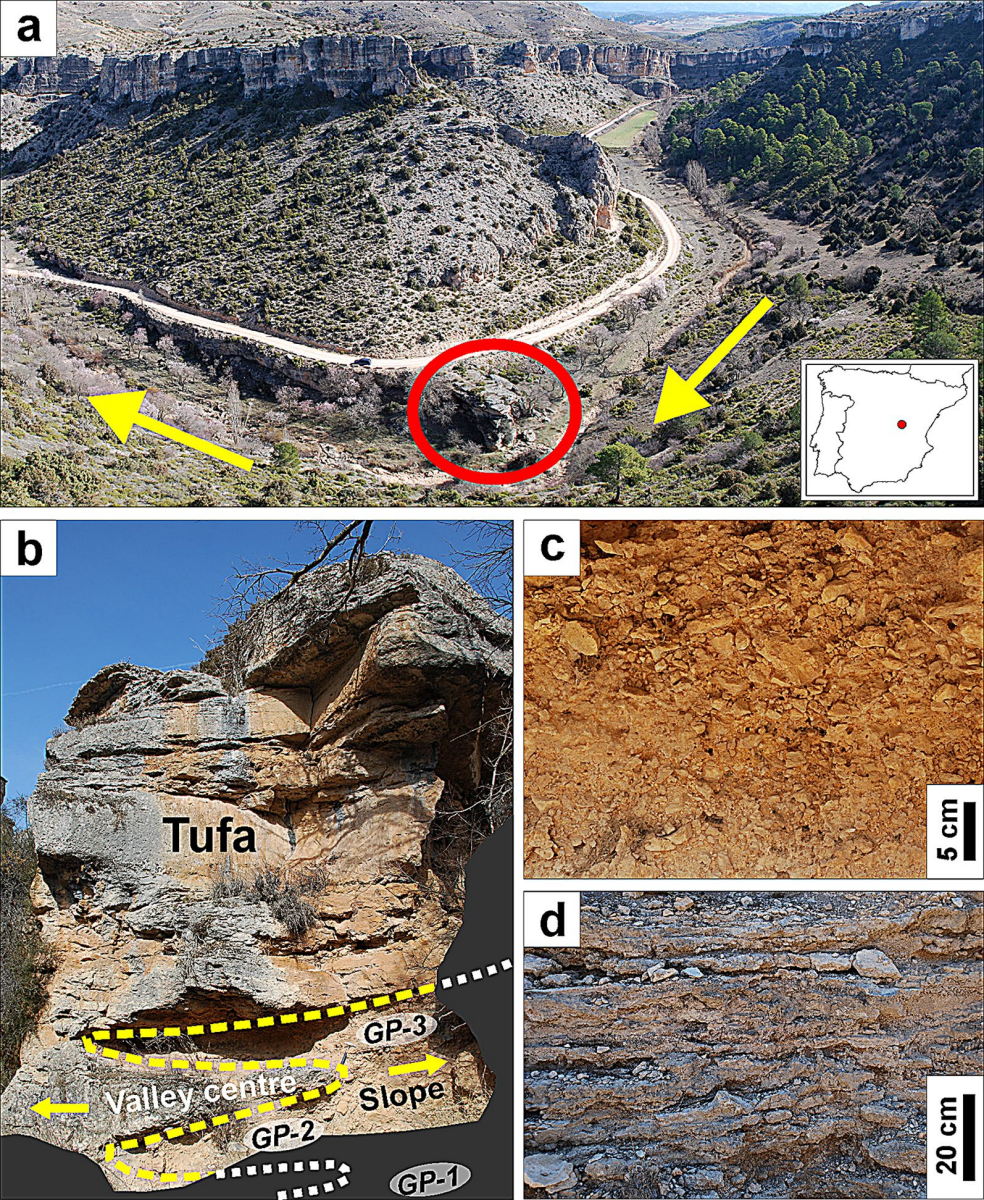
Pictures of Trabaque Canyon and the studied deposit. (**a**) Trabaque Canyon. The river flows according to yellow arrows. The red ellipse shows the location of the main section where the deposit was sampled. The inlet map shows the location of Trabaque Canyon within the Iberian Peninsula. (**b**) View of most of the studied Trabaque tufa section. The base and top of the section are missing from this panorama. The centre of the valley bottom is to the left of the image and the slope of the canyon to the right. The river flowed from the position of the observer towards the tufa deposit. The picture shows gravitational pulses GP-2 and GP-3 that interdigitate with the tufa deposit, and their disappearance from the bottom of the valley after GP-3. (**c**) Detail of GP-3 gravitational deposit. (**d**) Detail of the alternation between well-cemented and loose tufa beds at the top of the section.

Trabaque Canyon (40.36° N; 2.26° W; 840 m above sea level) is located in the central Iberian Peninsula (Fig. 1). At this site, tufa deposits precipitate as freshwater carbonates downstream of overflow karst springs. During the last interglacial period, tufa precipitated continuously at the studied site while water level of the aquifer was high enough for upstream springs to discharge^13^. Outcrops of the studied tufa deposit are preserved in the margins of Trabaque River over a distance of 500 m downstream of overflow karstic springs. The studied tufa deposit is 12 m thick, with a gentle ramp morphology, and a simple stratigraphy of sub-horizontal tufa beds that covered the full section of the narrow canyon. The accumulation of tufa created a small lake upstream the ramp, which prevented erosive events while the deposit was active, because most of the river bedload was accumulated in the basin of the lake. This configuration favoured the lack of erosive episodes in the tufa and the deposition of a continuous record. The tufa deposit was partially eroded by subsequent fluvial incision once the tufa accretion ceased and detrital sediments filled the lake basin and started to flow over the ramp during floods. The tufa deposit is mostly composed of well-cemented intra-clastic and peloidal carbonate particles^13^. The deposit comprises tufa beds 0.02–1 m thick that typically extend tens of metres downstream. At the base of the section, the tufa lies over loose fluvial sediments of sandy silt, whereas at the top of the section there is an erosive scar, and recent gravitational deposits overlay the tufa preserved in the slopes of the canyon.

**Figure 2:**
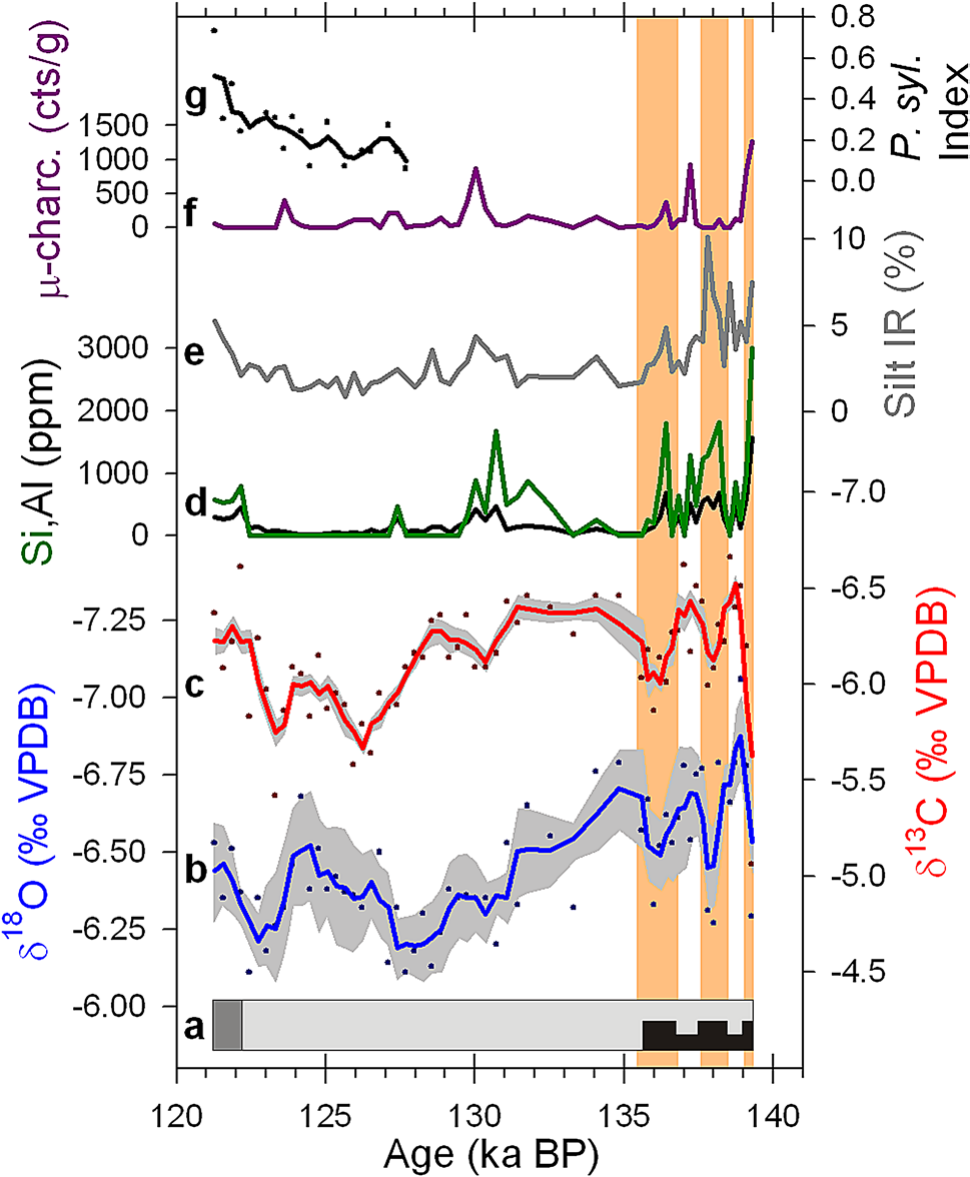
Record of the Trabaque tufa deposit. (**a**) Simplified lithological log of the Trabaque record. Patterns represent gravitational deposits (black) with distinct three pulses, well-cemented tufa sediments (light grey), and alternation of well-cemented and loose tufa sediments (dark grey). (**b**,**c**) Tufa δ^18^O and δ^13^C records. Isotope values at each date (dots) are the average of 3 sub-samples and blue/red line is a 3-point running mean. The grey shade shows the 1σ variability of the three sub-samples along the record. (**d**) Concentration of Si and Al. (**e**) Silt-sized insoluble residue in tufa as percentage of the total sample. (**f**) Counts of micro-charcoal particles < 125 μm per tufa gram. (**g**) *Pinus sylvestris* index shows the pollen ratio between *P. sylvestris/nigra* and the rest of dominant non-riparian arboreal trees. Vertical light orange bars show three pulses of dry and erosive conditions during the first stage of T-II.

The base of the deposit section is characterized by nearly 4 m of tufa sediments in the centre of the valley, laterally interdigitating with gravitational deposits towards the slopes (Fig. 1). These gravitational deposits partially invaded the bottom of the valley during three distinct pulses. These gravitational deposits occurred during periods of enhanced slope processes due to the decrease in vegetation cover on the canyon slopes during prolonged dry periods. The evidence of local erosion recorded by the gravitational deposits is consistent with other proxies that record local and regional erosion and that are displayed in Fig. 2. Thus, independent evidence of erosion is also recognized from the increase of insoluble residue (IR) particles in the tufa, recorded by the percentage of silt IR. IR particles were transported to the tufa by the river or by the action of wind. The increase of these particles in the tufa is interpreted as enhanced erosion, not only from the catchment but also from outside the basin. Higher concentrations of Si and Al are also interpreted as proxies of soil erosion from areas with silicate substrates inside or outside the catchment. The increase of micro-charcoal particles in the tufa is also interpreted as a sign of enhanced soil erosion. Charcoals were incorporated to the tufa during floods or transported by the wind after the occurrence of fires, as well as from the erosion of soils that accumulated charcoals from previous fire events. In any case, the increase of micro-charcoals in the tufa record suggests soil erosion due to the lost of vegetation cover. Major events of local and regional erosion occurred synchronously (Fig. 2), supporting that the common decreases in vegetation cover that resulted in erosion events were related to periods of reduced precipitation.

The middle section of the tufa deposit (i.e., 4–11 m from the base), does not record gravitational deposits in the centre of the valley. In agreement with this retreat of the gravitational deposits to the slopes of the canyon, other proxies of erosion record minimum values along most of the middle section of the tufa. This supports the expansion of forest and/or vegetation cover in the region, which protected soils from erosion. The top section of the tufa (i.e., 11–12 m from the base) records an alternation of well-cemented and loose tufa beds (Fig. 1). The loose beds were formed when karst groundwater discharge upstream of the studied site occurred as overflow instead of permanent springs, as a response to a drop in the level of the aquifer^14^, which limited the formation of cements. The decrease in precipitation that caused this drop of the groundwater level was also responsible for the progressive increase in percentage of *Pinus sylvestris/nigra*, a tree with high tolerance to cold and dry conditions (Fig. 2). Therefore, the tufa deposit records periods of significant hydrological deficit in the system at the base and the top of the section.

The tufa δ^18^O and δ^13^C values have a clear response to climate during the early phase of deglaciation, while forest cover in the Iberian Peninsula was variable. However, once the forest cover expanded in the Iberian Peninsula^15^, additional factors in the water and carbon cycles complicated the link between climate and these proxies. The δ^18^O oscillations recorded at the base of the section, are interpreted as changes in the amount of precipitation (i.e., more negative δ^18^O values were recorded with increased precipitation). During this period, amount of precipitation and the ratio of recycled precipitation, two main controls on the δ^18^O values of precipitation in the Iberian Peninsula^16^, co-varied due to the positive feedback between precipitation and forest coverage, which determined the ratio of recycled precipitation. Once forest expanded in most of the Iberian Peninsula, limited changes in forest cover caused the ratio of recycled precipitation to be independent of the amount of precipitation, and recycled precipitation instead of amount of precipitation dominated the δ^18^O signal^17^. Temperature is not a significant control on Trabaque tufa δ^18^O values at inter-annual timescales, because of isotope fractionation at the time of raindrops formation and atmosphere equilibration is counteracted by a similar isotope fractionation of opposite sign when calcite precipitates^18,19^. On the other hand, the large δ^13^C oscillations recorded at the base of the record were controlled by the variability of CO_2_ degassing (i.e., more negative δ^13^C values occurred during periods of increased precipitation that raised the level of the aquifer and limited the air space for degassing). Once the forest cover stabilized in the catchment area, enhanced soil activity increased the importance of biological controls on the carbon cycle and limited an unequivocal interpretation of the δ^13^C signal. The base of the tufa deposit records three δ^18^O and δ^13^C oscillations that represents periods of drier and wetter conditions. Drier conditions according to the isotope records occurred at the time of enhanced local and regional erosion recorded in other Trabaque proxies. The replication of climate and environmental signals from multiple proxies supports the robust interpretation of the record.

A chronological study based on radiometric dates from this deposit confirmed that the tufa was mostly formed during the last interglacial period^13^, although its uncertainty prevents discernment of the age of events at a millennial timescale. Since the morpho-stratigraphical evidence supports that Trabaque sequence records a continuous time series, we use the age model of Corchia speleothems, located in central Italy^8^ to improve Trabaque chronology. This synchronization was conducted using δ^18^O anomalies that are clearly identifiable in both records (Supplementary Figs. S1–Fig. S4; supplementary Table S1). We compare the Trabaque record to a key ocean sediment core (ODP 984) located south of Iceland (61.25° N; 24.02° W) since this location is particularly sensitive to record ice rafted debris (IRD) events during the deglaciation^11^. We also synchronize the age model of ODP984 record to the Corchia chronology (Supplementary Figs. S5–S6; supplementary Table S1), based on the strong connection between the Mediterranean climate and the North Atlantic during periods of ice-sheet instability^20^.

### First stage of T-II

**Figure 3:**
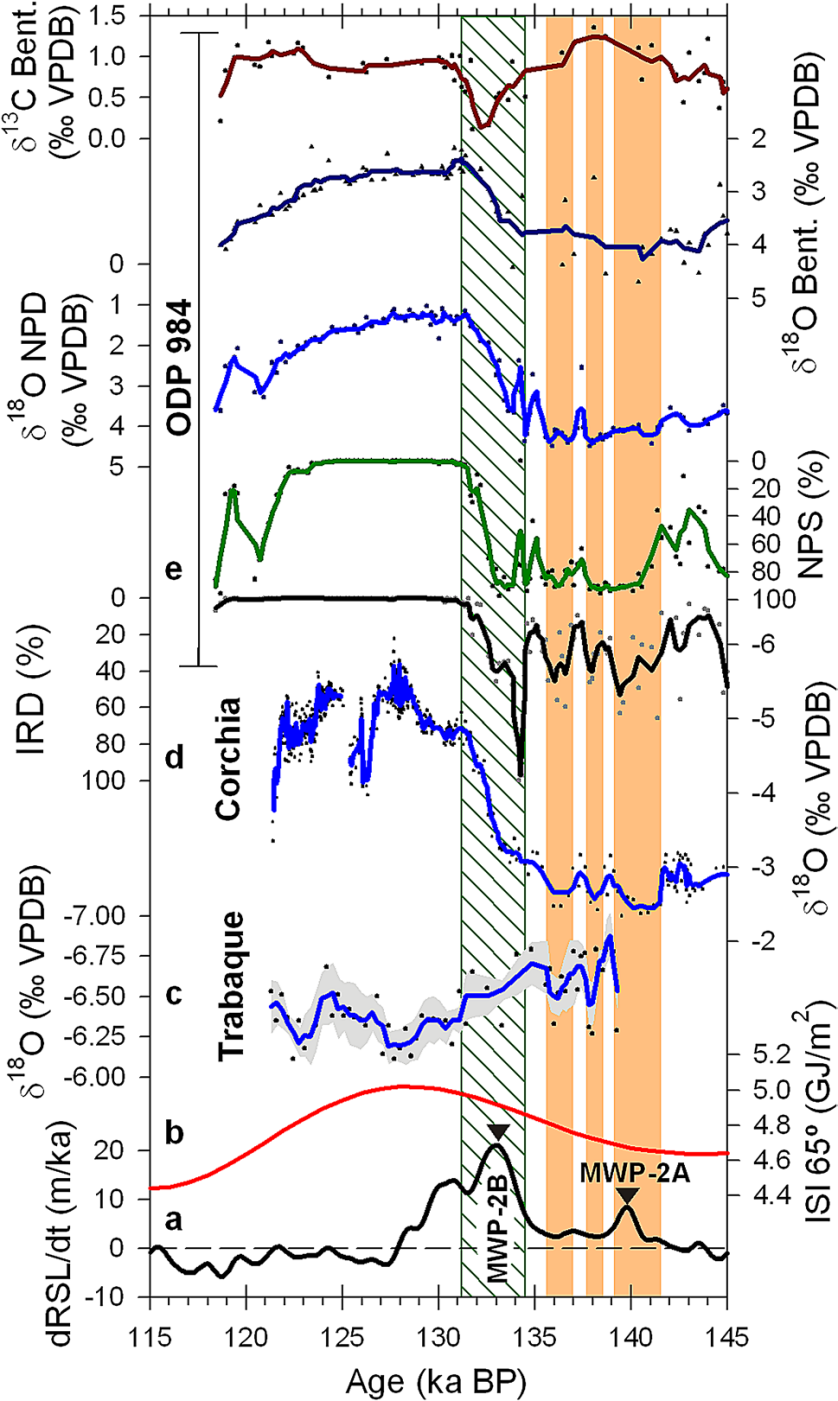
North Atlantic and Mediterranean records during T-II. (**a**) Rates of relative sea-level change (dRSL/dt)^6^. Curve above the horizontal dashed line represents sea level rise, and relative maxima show particular periods of increased sea level rise. Meltwater pulses MWP-2A and MWP-2B are indicated with triangles. (**b**) Integrated summer insolation (ISI) at 65 °N^7^. (**c**) Trabaque tufa δ^18^O record as shown in Fig. 2. (**d**) Corchia speleothem composite δ^18^O record^8^. Dots are raw data and blue line is the 5-point running mean. (**e**) Records of IRD percentage, NPS percentage, planktonic δ^18^O values, benthonic δ^18^O values and benthonic δ^13^C values from the ocean core ODP 984, South of Iceland^11^. Dots represent raw data and lines are 3-point running means. *IRD* Ice rafted debris, *NPS* *Neogloboquadrina pachyderma* sinistral cooling. Vertical light orange bars show three pulses of dry and erosive conditions during the first stage of the T-II. Vertical striped bar shows the period of collapsed Thermohaline Circulation indicated by less positive benthonic δ^13^C values.

During a first stage of T-II, three millennial oscillations are recorded in the North Atlantic and the Mediterranean (Fig. 3). In Corchia Cave, less negative speleothem δ^18^O values during these oscillations represent drier conditions^8^, that correspond with dry and erosive conditions in Trabaque record, and the deposition of IRD events at ODP 984 site. According to an independent synchronized chronology^6^, the first meltwater pulse of the T-II (MWP-2A), occurred during the cold/arid period of the first millennial oscillation, at the time when the integrated solar insolation in the Northern Hemisphere started to rise^7^. The initial ablation of Northern Hemisphere continental glaciers provided freshwater to the ocean surface and enhanced the halocline in the high latitudes of the North Atlantic, while the North Atlantic Current still flowed into the Nordic Seas^10^. The sharp salinity gradient in surface waters of the Nordic Seas caused salty waters from the North Atlantic Current to flow under the halocline, preventing the release of heat from those relatively warm waters to the atmosphere. This decoupling of the North Atlantic Current from the atmosphere enhanced the cold conditions in the high latitudes of the Northern Hemisphere, triggered the formation of ice rafted debris (IRD) events in the North Atlantic^21^, and the related decrease of precipitation in the Mediterranean region. Two periods of enhanced glacier ablation recorded south of Greenland^22^, are likely related to episodes of enhanced IRD deposition at ODP 984 site subsequent to MWP-2A and arid/erosive conditions recorded in the Mediterranean region. The cold climate in the Nordic Seas, caused by the decoupling of the North Atlantic Current from the atmosphere, led to a negative feedback that progressively reduced the rate of glacier ablation and eventually disrupted the stratification of surface ocean waters. Without a significant stratification in the Nordic Seas, heat was released from the North Atlantic Current to the atmosphere in the region, which enhanced the ablation of continental glaciers and increased the input of freshwater to the ocean. These interactions resulted in climate oscillations at millennial scale. During this first stage of the deglaciation, no shutdown of the Thermohaline Circulation was recorded^23^. The mechanism causing these millennial climate oscillations clearly originated from modifications of the ocean–atmosphere interactions at high latitude of the North Atlantic. The impact of these anomalies in the ocean circulation seems to be limited to the uppermost section of the water column as recorded by planktonic assemblages^11^, while deep or intermediate waters kept their flow^10^. Therefore, these anomalies were not propagated by the ocean circulation to the Southern Hemisphere^23^ and consequently they differ from other millennial scale climate interactions that involve the BSM such as Heinrich or Dansgaard-Oeschger events.

### Second stage of T-II

The second stage of T-II started with the onset of a fourth IRD event recorded at ODP 984 site at 134.5 ± 0.9 ka BP. This IRD event, known as Heinrich event 11 (H11)^24^, was much larger than previous IRD events of T-II, although its onset was still in phase with the pace of the millennial oscillations. H11 significantly contributed to the larger meltwater pulse within T-II (MWP-2B)^6^. However, the glacier decay that resulted from the progressive increase of integrated summer insolation in the Northern Hemisphere^7^ was disproportionate to the magnitude and duration of H11 and MWP-2A, even accounting for the enhanced freshwater supplied to the North Atlantic by ice-sheet decay that resulted from the millennial oscillations. An event or a surpassed threshold in deglaciation occurred that triggered the observed non-linear response. Ocean sediments from Bermuda Rise recorded an increase in the flux of clay from Canada just before the shutdown of the Thermohaline Circulation^25^. This suggests a significant outburst from North American proglacial lakes, a scenario supported by climate models^26^. The outburst occurred in phase with the enhanced Northern Hemisphere ice-sheet decay related to the millennial oscillations, which controlled its timing. The large amount of freshwater released to the North Atlantic in relation to the H11 caused the collapse of Thermohaline Circulation^23^ and triggered the BSM that forced the Southern Hemisphere to react for the first time since the onset of T-II.

The activation of the BSM initiated the second stage of T-II. The erosion in the Trabaque catchment during H11 was limited, especially during the first half of this cold period (Fig. 2; Supplementary Fig. S7). So, unlike previous IRD events, H11 was not a particularly dry event in the Iberian Peninsula. During H11, Trabaque deposit recorded its lowest growth rate (Supplementary Fig. S4). At this time, the North Atlantic off the Portuguese margin had a winter temperature drop of 6 °C^27^. The continental and mountainous location of Trabaque Canyon results in nowadays mean temperature of the coldest month (December) < 3 °C. Therefore, the drop of temperature during H11 likely resulted in seasonal freezing of the lake surface upstream the studied site, and limited the growth rate of tufa during the H11.

**Figure 4:**
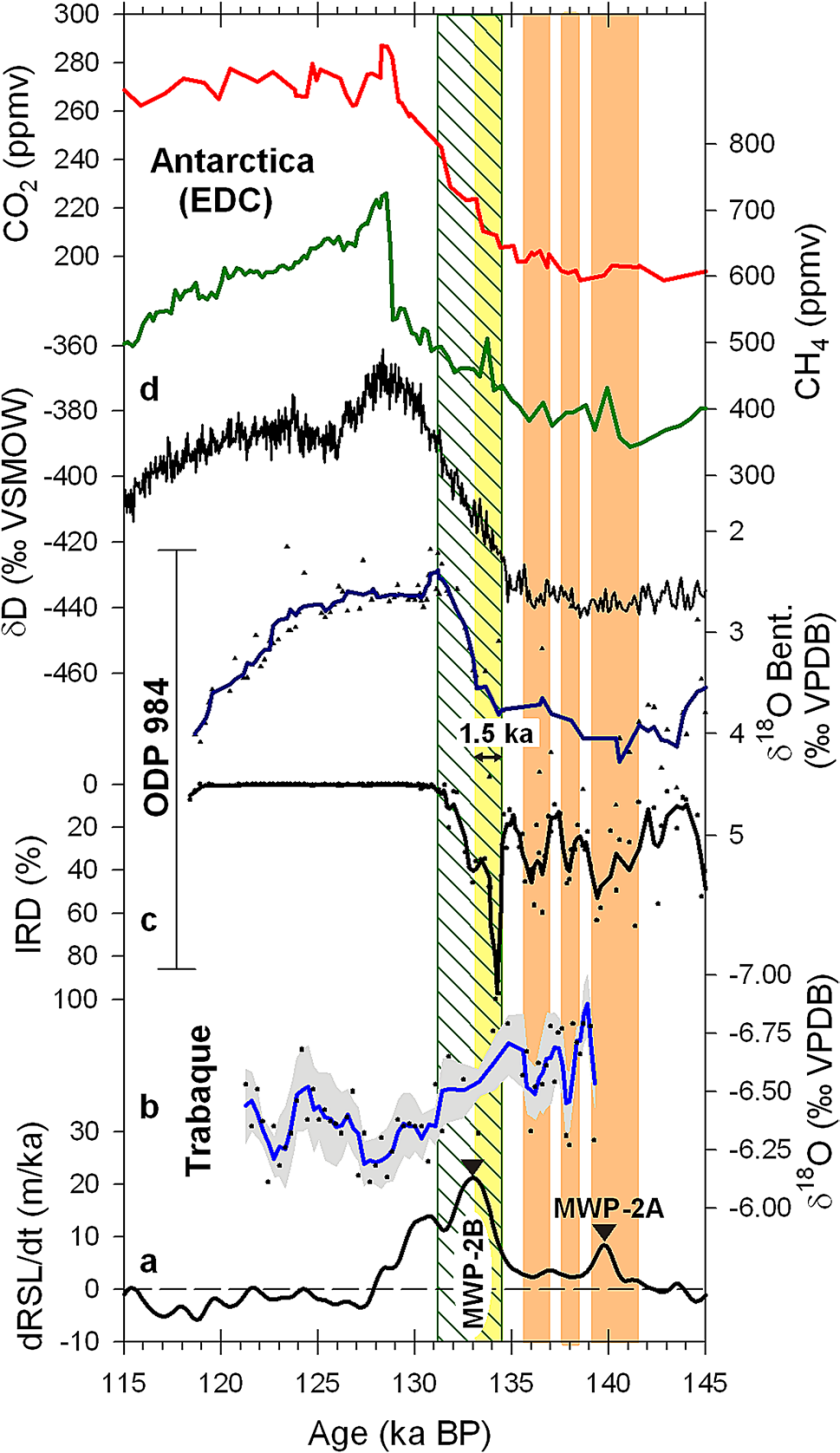
Bipolar Seesaw Mechanism during T-II. (**a**) Rates of relative sea-level change (dRSL/dt)^6^ and meltwater pulses MWP-2A and MWP-2B shown as in Fig. 3. (**b**) Trabaque tufa δ^18^O record shown as in Fig. 2. (**c**) Records of ice rafted debris (IRD) percentage and benthonic δ^18^O values from the ocean core ODP 984, South of Iceland^11^. (**d**) Records of δD and CH_4_ from the Antarctic ice core EDC^28,42^ and CO_2_ from multiple polar ice cores^43^. The ice core chronology is on the AICC2012 timescale^44^. Vertical light orange bars show three pulses of dry and erosive conditions during the first stage of T-II. Vertical striped bar shows the period of collapsed Thermohaline Circulation. Vertical light yellow bar shows the lag time (1.5 ± 0.8 ka) between the onset of the collapse of the Thermohaline Circulation and the invasion of Antarctic Bottom Waters at the ODP 984 site, indicated by less positive benthonic δ^18^O values.

The suppression of the Thermohaline Circulation caused the expansion of the Antarctic Bottom Water through the North Atlantic basin^23^. Temperature in Antarctica^28^ started to increase once the BSM was activated, and ablation of glaciers from the Southern Hemisphere contributed in part to the MWP-2B (Fig. 4). The freshwater released around Antarctica enhanced wind activity and caused a northward shift of the Antarctic Polar Front that favoured the upwelling of deep water from the Southern Ocean and the release of CO_2_ to the atmosphere^12,29^. When H11 finished, Northern Hemisphere continental glaciers outside Greenland were mostly ablated, MWP-2B ended, the Thermohaline Circulation was re-established and most of the Northern Hemisphere had a climate typical of an interglacial period^2,23,30^. At this time, Trabaque tufa recorded limited regional erosion because forest dominated the landscape (Supplementary Fig. S8). As a result of the BSM, the deglaciation in the Southern Hemisphere continued during two more thousand years before T-II was completed.

### The role of millennial climate oscillations on terminations

Previous studies have pointed out that millennial oscillations probably played an important role in terminations^5,31^. The occurrence of H11 was in-phase with the millennial oscillations that dominated the climate of the North Atlantic and the Mediterranean, which constrained the time frame for the trigger of the BSM. The climate interactions that operated during the first stage of T-II, (i.e., millennial oscillations of heat release to the atmosphere at high latitudes of the Northern Hemisphere from the North Atlantic Current), were not observed during Termination I (T-I)^30^, because of North Atlantic Current did not flowed into the Nordic Seas during T-I^10^ and the rates of insolation change were very different^7^. The heat transported by the ocean into high latitudes triggered the millennial climate oscillations recorded during the first stage of T-II as a result of the coupling/decoupling of ocean–atmosphere heat fluxes in the Nordic Seas. This particular climate mechanism was absent during T-I, even if the structure of T-I was also dominated by millennial climate oscillations (e.i., Mistery Interval-Bølling/Allerød-Younguer Dryas). The millennial oscillations of T-I were controlled by different climate mechanisms and resulted in an early BSM that caused both hemispheres to respond synchronously at a millennial timescale^32,33^. The sequence of events and the evolution of the sea level rise clearly differed in the last two terminations, which is reflected in their structure, duration and timing^23,30,34,35^. Thus, together with the variable rates of insolation change, the climate mechanisms that control the millennial oscillations could account for the different structure observed between T-I and T-II^2^. The structure of T-II is very similar to other terminations such as T-IV, T-V or T-VI^9,36^, suggesting that the operation of millennial oscillations controlled by similar climate mechanisms as during the first stage of T-II could have occurred during those deglaciations. Therefore, we suggest that the difference in structure between terminations and the precise timing of the major events in the deglaciations could be controlled not only by the rates of insolation change, but also by the climate mechanisms behind the millennial oscillations within the terminations, which characterizes different modes of deglaciation.

### Inter-hemispheric asynchrony of stages during T-II

Description of two stages or pulses were previously reported during T-II^6,10,34,35,37^, although these stages are defined by different events and consequently they are often not equivalent stages. We define two stages during T-II easily identified in marine and continental records of the North Hemisphere. The duration as well as the onset and demise of T-II were not synchronous in both hemispheres. The Southern Hemisphere only recorded the second stage of T-II, and the clear evidence of regional deglaciation started 7 ka after the onset of T-II. However, the end of deglaciation during T-II lasted 2 ka more in the Southern Hemisphere, while in the Northern Hemisphere full interglacial conditions were already established. The sequence of events here reported shows that the first stage of T-II was initiated with the progressive decay of the large ice-sheets in the Northern Hemisphere responding to the increase in summer insolation. However, the deglaciation did not occur at a steady rate, and complex ocean–atmosphere interactions in the high latitudes of the Northern Hemisphere caused millennial climate oscillations that paced the decay of ice-sheets. These millennial oscillations controlled the timing of a large glacier outburst that triggered the H11, and the collapse of the Thermohaline Circulation that initiated the BSM at the onset of the second stage of T-II. Although most drastic deglaciation events occurred with the onset of the second stage of T-II, the termination already started 7 ka earlier. To understand the complete sequence of events that enabled the completion of T-II, it is essential to cover not only the most significant changes of the deglaciation, but also those smaller oscillations that eventually were responsible of triggering the larger climate changes.

## Conclusions

We identified two stages during the evolution of T-II. The first stage of T-II is recorded in continental and oceanic sites and is characterized by three millennial climate oscillations. However, this stage is only recorded along the Northern Hemisphere. The second stage of T-II was initiated along with the large Heinrich event H11 that coincides with an outburst of proglacial lakes from North America that triggered the BSM and initiated the deglaciation in the Southern Hemisphere. The pace of the millennial climate oscillations during the first stage of T-II controlled the precise timing of the BSM that was responsible for the larger deglaciation events recorded worldwide. Terminations often record millennial oscillations that control the structure of the deglaciations, although the mechanisms that cause those oscillations are not necessarily equivalent. During the first stage of T-II warm subtropical waters flowed into the Nordic Seas, whereas during T-I those waters never reached such high latitudes. We suggest that millennial oscillations are key to understand the structure and precise timing of events during deglaciations and that different climate mechanisms controlling those millennial oscillations result in different modes of deglaciation.

## Materials and methods

### Tufa sampling

To establish a precise depth scale, samples were collected using horizontal cores 0.025 m in diameter and up to 0.15 m in length along the vertical section of the tufa deposit at 0.2 m intervals. The external end of each core (typically ~ 0.01 m) was discarded to avoid potential contamination of the original tufa due to carbonate weathering or precipitation of carbonate crusts.

### Carbon isotope analyses of organic matter from tufa carbonates (δ^13^C_org_)

Tufa samples of approximately 0.5 g were dissolved in 2 M HCl to eliminate carbonates, and the solutions were evaporated in a sand bath at 50 °C. The organic residue of each sample was analysed three times using a Europa 20–20 continuous flow IRMS coupled to an ANCA SL elemental analyser. Analyses were repeated when the standard deviation of replicates was > 0.2‰. The reported δ^13^C_org_ values are the average of the three analyses of every sample and their uncertainty was 0.2‰. The analyses were carried out to constrain the role of δ^13^C values from organic fraction (δ^13^C_org_) on the aqueous carbon isotope composition that eventually affected the tufa carbonate δ^13^C values.

### Oxygen (δ^18^O) and carbon (δ^13^C) stable isotope analyses of tufa carbonates

Three independent sub-samples were analyzed from each tufa core. Thus, 3 complete runs of the full section are available. The samples were analysed in a continuous flow Isoprime IRMS and uncertainties were 0.11‰ for δ^18^O values and 0.04‰ for δ^13^C values. The isotope composition of tufa at particular depths was calculated as the mean of the three available analyses. The reported average isotope values reduce the inter-sample variability caused by the heterogeneous composition of samples within the core and/or the analytical uncertainty, and are more representative of the average isotope composition of the tufa at inter-annual timescales. To enhance the millennial component of the isotope record, the isotope signal was filtered with a 3-point running mean.

### Insoluble residue (IR)

From each core, a tufa fragment (50–120 g) was dried, weighed and dissolved in 18% HCl. The solution was filtered and the recovered insoluble residue (IR) was dried, weighed and reported as percentage of IR in relation to the total tufa sample weight. Clay and fine silt fractions (< 30 μm) were eliminated with the solution and not accounted as IR. The residue samples were wet sieved through mesh sizes of 250, 125 and 63 μm to evaluate the texture of the IR. No particle larger than 500 μm was recorded. Silt fraction IR is on average 98% of the total IR.

### Trace elements

Concentrations of Si, Al, Mg and Sr were measured with ICP-MS after the dissolution of ca. 2 mg of sample in HNO_3_. Calibration curves were constructed with standard solutions at five different concentrations. Standards and samples were spiked with known concentrations of Sc, Y and In. The recovery of these tracers was > 95 ± 5%. Mg and Sr are reported as Mg/Ca and Sr/Ca weight ratios respectively, since these elements are important in the carbonate system and allow the evaluation of dissolution and precipitation of carbonates. Ca was estimated based on the amount of dissolved tufa sample corrected for the non-carbonate proportion measured by the total IR.

### Pollen and charcoal

For each sample, approximately 5 g of well-cemented tufa was dissolved in 37% HCl to eliminate carbonates. Standard pollen extraction techniques were applied to processed samples^38^, although acetolysis was not carried out to allow the identification of any contamination by modern pollen. Densimetric extraction of pollen and charcoal particles was done with Thoulet solution and the processed samples were mounted in glycerine for the identification of pollen palynomorphs under microscope at 400× and 1,000× magnification by the use of keys and photo atlases. Identification of *Pinus pinaster* pollen type followed criteria by Carrión et al.^39^. A minimum of 300 grains of total land pollen (TLP) per sample were identified and counted. Percentages were calculated based on TLP sum excluding hydro-hygrophytic taxa and non-pollen palynomorphs. Charcoal debris was counted under microscope, along with the identification of pollen, and classified by size. Charcoal particles > 125 μm are related to fires within the catchment area, whereas charcoal particles < 125 μm, or micro-charcoal particles, can also result of fires from more distant sources^40^. Large charcoal particles can be broken in smaller pieces during the sample preparation process. However, since the same protocol was applied to all samples, we assume that any bias was systematic and the variability within the record was not affected. Abundance of charcoal was reported as number of particles per gram of sample.

### Synchronization of records

A radiometric age is available for the studied Trabaque tufa deposit (120.2 ± 8.0 ka BP), which was calculated using a U-Th isochron obtained from multiple samples collected 1 m from the top of the section^13^. In addition, sedimentological and morpho-stratigraphical data that accompanied the chronological study confirmed that the tufa section had continuous deposition during thousands of years around the last interglacial. However, because of the large uncertainty, the age of the tufa section cannot be constrained with more detail based on the radiometric age. Instead, we use the clear structure of the Trabaque tufa δ^18^O record in relation to the onset of the last interglacial period (Supplementary Fig. S1) to synchronize our record with a better constrained chronology. We choose the speleothem record from Corchia Cave^8^ that is correlated with other outstanding records in the western Mediterranean region^23^. The composite Corchia chronology, based on two overlapping speleothems was used to minimize the bias related to individual samples. Trabaque Canyon and Corchia Cave are under the same area of climatic influence, and major changes in the hydrological cycle that affect the δ^18^O of precipitation in both sites are expected to occur simultaneously. Climate and/or environmental changes recorded in the tufa δ^18^O signal were synchronously recorded in other proxies from the Trabaque record. So, although data for synchronization was taken from the tufa δ^18^O record, occasionally other proxies were also used to support the decision on the exact timing of climate or environmental events when the δ^18^O variability was limited. The amplitude of Trabaque δ^18^O record is limited and secondary controls may modify the net direction of anomalies when major reorganizations affect the hydrological cycle (e.g., changes in global ice-volume affecting the δ^18^O of precipitation). So, we set our tie points (TP) in transitional periods of the δ^18^O series or in short lasting and well-defined events, while we used paleoclimate and paleoenvironmental oscillations interpreted from multiple proxies to confirm that correlations are not phase shifted during millennial oscillations. It should be noticed that when the major climate changes recorded in multiple proxy records from Trabaque and Corchia sites are aligned, the δ^18^O anomalies between both records show a non-stationary relationship through time, even if changes in the δ^18^O signal occurred synchronously. We also create an age model for the ocean record from the ODP 984 site. We selected indicators of the ocean surface waters, the percentage of ice rafted debris (IRD) and/or *Neogloboquadrina pachyderma* sinistral cooling (NPS) records, to synchronize the ODP 984 record to the δ^18^O Corchia speleothem record. Detailed description of TP is described in the supplementary information.

### Age models and calculation of the uncertainty

The synchronization of records is inspired by similar methods^6,30,41^. The Corchia Cave chronology, originally reported as ka B2007, was transferred to ka BP scale. For every TP we calculated 1,000 random dates following a normal distribution according to the Corchia age and its 2σ uncertainty. The average of the Monte Carlo simulations for every TP was selected as the anchoring date for Trabaque and ODP 984 records. Selected anchoring dates and Corchia dates for the TP (both in ka BP scale) have a maximum difference of 0.03 ka. The age model was calculated using the linear interpolation method between anchoring dates to preserve the synchrony with Corchia δ^18^O record^30^. According to the age model, the growth rate of Trabaque record is between 1.13 and 0.26 m/ka. This range of accumulation rate is common in tufa deposits. On the other hand, the average growth rate calculated from the age model of ODP 984 record is 0.27 m/ka, which is common for high accumulation rate sites in the North Atlantic. The uncertainty of the age model was calculated from the square root of the sum of the squares of three sources of uncertainty: (1) the transfer of the Corchia age model uncertainty to the synchronized records, (2) the temporal resolution of the synchronized record, and (3) the temporal resolution of Corchia record. Most of the uncertainty is related to the first source of uncertainty. Linear equations from contiguous and alternate anchoring dates were used to construct 1,000 simulated dates for every depth of the synchronized records. The dates were determined as the average of results from all equations available at each depth. Thus, the first source of uncertainty is calculated as the 2σ of the 1,000 simulated dates at every depth. The second and third source of uncertainty accounts for the chronological errors associated with the definition of every TP due to the temporal resolution between samples in the Trabaque, ODP 984 and Corchia records.

## Supplementary information

Supplementary information.
