# Supplementary material for: Millennial climate oscillations controlled the structure and evolution of Termination II
Source: Sci Rep. 2020 Sep 10;10:14912. doi: 10.1038/s41598-020-72121-4 (PMC7484808; doi:10.1038/s41598-020-72121-4)
Supplement: Supplementary file 1 — Supplementary information. [file 41598_2020_72121_MOESM1_ESM.pdf]

Supplementary information

Millennial climate oscillations controlled the structure and evolution of Termination II

David Domínguez-Villar, Juan A. Vázquez-Navarro, Kristina Krklec, Sonja Lojen, José A. López-Sáez, Miriam Dorado-Valiño & Ian J. Fairchild

Description of tie points used in the synchronization

The synchronization requires the selection of tie points (TP) in both records. The chronology of Corchia Cave speleothems[1] was used to synchronize Trabaque record. We identified clear anomalies recorded in the $\delta^{18}O$ signals of both, Corchia speleothems and Trabaque tufa, and the speleothem dates for the selected TP were transferred to Trabaque record to construct its chronology. The Corchia Cave $\delta^{18}O$ signal records three millennial oscillations before the larger changes of the T-II (Supplementary Fig. S2). These three oscillations are clearly recorded in most proxies from Trabaque, including its $\delta^{18}O$ record. We chose the midpoint of the transitions at the end of each $\delta^{18}O$ millennial oscillation (TP1, TP2, and TP3). A common signal in many oceanic and continental $\delta^{18}O$ records is a small and short-lasting reversal event in the middle of the isotope transition between the glacial and the interglacial periods. We selected the central peak of this event (TP4). A characteristic event clearly discernable in most ocean and some continental archives is the first cold event (C28 in ocean terminology) that occurred soon after the completion of the T-II in the Northern Hemisphere. Despite the climate importance and wide recognition of this event in paleoclimate records, the variability of the $\delta^{18}O$ signals in Corchia and Trabaque records is limited. So, as a single exception, we use proxies other that $\delta^{18}O$ to define a TP in this significant climate event (TP5). Silt IR and microcharcoals among other proxies from Trabaque record respond clearly to this event, whereas the planktonic $\delta^{18}O$ record from the ocean core ODP 977A, that was previously synchronized to Corchia speleothem $\delta^{18}O$ record[1], clearly identifies the timing of the event in Corchia Chronology. Finally, we selected the mid point of the $\delta^{18}O$ transition at the end of the optimal conditions of the interglacial period (TP6).

The chronology of Corchia speleothems was also used to synchronize the subpolar ocean sediment record from the site ODP 984[2]. In this case, we match clear anomalies of the $\delta^{18}O$ record of Corchia speleothems (unless for TP5 as previously explained) with equivalent anomalies of either the IRD record or the percentage of *Neogloboquadrina pachyderma* sinistral cooling (NPS) record of the ocean sediments. We selected the same TP as those used for Trabaque record, although one additional TP was included at the base of the record (TP0) to account for its longer duration. The middle of the transition at the onset of the first millennial oscillation recorded in the Corchia $\delta^{18}O$ signal was selected for TP0. The proportion of IRD recorded large variations during the deglaciation period and was preferred to identify every TP during the T-II, whereas the percentage of NPS record was more sensitive to changes during the interglacial period and we used it to identify TP5 and TP6 (Supplementary Fig. S5). Exact dates of TP used in the synchronization are reported in the supplementary Table S1.

Additional proxies studied in Trabaque record

To avoid the display of proxies that show a repetitive pattern or proxies that were used to constrain controls on key proxies, only a selection of the proxies studied in Trabaque record are presented in the main text and Fig. 2 of this manuscript. Next we describe the records of the proxies not previously reported and discuss their contribution to the climate and environmental interpretation of Trabaque record. The complete set of proxies studied in Trabaque record is displayed in supplementary figures S7 and S8.

Carbon isotope analyses of organic matter from tufa carbonates ($\delta^{13}C_{org}$). There is a lack of significant correlation between $\delta^{13}C_{org}$ and $\delta^{13}C$ records (Supplementary Fig. 7). Therefore, we consider that, despite the 4 ‰ range recorded in the δ^{13}Corg values, the variability of tufa carbonate $\delta^{13}C$ values was not dominated by changes in the carbon isotope composition of the organic matter. However, these data confirm that vegetation with C3 photosynthetic pathway dominated the catchment area during the period of tufa deposition.

Sand-fraction insoluble residue (IR). The morphology of the tufa deposit favoured a relative limited content of non-carbonate sand fraction. Only particles transported by suspension or some of those transported by saltation were recorded in the tufa ramp beyond the sedimentary basin of the lake upstream the tufa. Recorded sand particles were not only transported by the river, since aeolian activity could also be an efficient agent of transport. In any case, we consider that the increases in the percentage of sand IR in tufa samples mostly reflects enhanced erosion in the catchment area. On the other hand, the increase of silt IR may reflect erosion not limited to the catchment area, because of the facility of silt particles to be transported from remote areas by aeolian processes. Thus, part of the silt IR fraction may originate from different biogeographical areas, where erosion processes may differ from those in the proximity to Trabaque Canyon. Sand and silt IR signals are very similar during most of the record, with two outstanding exceptions.

(1) The percentage of sand IR in tufa is higher in the 4 m from the base of the record, and shows sharp changes (Supplementary Fig. 7). In agreement with other proxies showing erosion, sand IR increases during the three arid and erosive periods recorded at the base of the Trabaque section. In addition, a significant peak in sand IR values is also recorded outside these arid periods. In this case, it is clearly related to an increase in charcoals, and likely resulted from enhanced erosion after a fire within catchment area during a relatively wet period. During this anomalous event of higher percentage of sand fraction in tufa, there is no significant increase in the silt IR signal, which supports the local and not climate related origin of the recorded erosion.

(2) At the top of Trabaque section, another clear divergence is observed between sand and silt IR signals (Supplementary Fig. 7). In this case, there is a progressive increase of the percentage of silt IR in the tufa, whereas the sand fraction continues its low values. This divergence suggests that erosion in the vicinity of Trabaque was limited, while increased in more distant areas. Pollen record supports that at this time the landscape around Trabaque was dominated by a dense forest cover supporting the lack of local erosion. However, proxies such as the *Pinus sylvetris* index or the Mg/Ca and Sr/Ca ratios clearly support a climate progressively drier during this period. These dry conditions likely decreased the vegetation cover in different biogeographical regions in the interior of the Iberian Peninsula and enhanced erosion processes providing a source for the silt recorded in Trabaque Canyon at this time.

Tufa Mg/Ca and Sr/Ca ratios. Si and Al concentrations reflect partial dissolution of silicate particles in the tufa. Hence, we interpret that the increased concentration of these elements results from larger proportions of silicates in the tufa, caused by enhanced erosion in the region. Partial dissolution of silicates in the tufa results in higher values of elements contained in silicates. Together with Si and Al, Mg is also a common element in silicates, and coeval concentration peaks of Si, Al and Mg implies that the Mg signal originates mostly from the presence of abundant silicates in the sample. Such origin of Mg is observed mostly at the base of Trabaque section (Supplementary Fig. 7), although some carbonates, such as dolomites, also contain important concentrations of Mg. In carbonate systems, the proportion of Mg and Sr in relation to Ca (i.e., Mg/Ca and Sr/Ca ratios) are useful to understand dissolution and precipitation processes. Precipitation of calcite cements within the aquifer (i.e., prior calcite precipitation), result in the decrease of Mg/Ca and Sr/Ca ratios in the solution[3]. This hydrochemical change is recorded in subsequent precipitates of calcite, such as the tufa sediments, that often occur when karst groundwater emerges to the surface. The monitoring of the hydrochemistry of Trabaque system showed that the common ion effect, enhanced by the dissolution of gypsum in the catchment area, favours the precipitation of calcite cements within the aquifer when dolomite is dissolved as part of a dedolomitization process[4]. This dedolomitization process was enhanced when water table levels were low. Therefore, we interpret that the decrease in Mg/Ca and Sr/Ca ratios, as recorded at the top of Trabaque tufa section, suggests a drop of the water table level. This is in agreement with other proxies from the record that supports a trend towards a more arid climate at the top of Trabaque record.

Charcoal and Pollen. Charcoal particles were successfully recorded along the full Trabaque section. The size of charcoal particles was considered during its counting (i.e., micro-charcoals were <125 μm). However, there is little difference between both charcoal records (Supplementary Fig. 7). On the other hand, preservation of enough pollen to provide a reliable record was only possible in the upper part of Trabaque section (Supplementary Fig. S8). The pollen record shows that a typical Mediterranean forest dominated the landscape around Trabaque Canyon from at least 128 ka BP and until the end of the tufa section. Multiple proxies from Trabaque tufa sediments record limited erosion coeval with the presence of the Mediterranean forest. The low levels of erosion recorded in Trabaque tufa commenced at 135.5 ka BP, likely as a result of the expansion of the forest coverage not only in the catchment area but in a wider region. At the base of the section, multiple proxies from Trabaque record indicate the existence of three major periods of aridity accompanied with local and regional erosion. These periods of erosion suggest that in agreement with other Southern European sites[5-7], the expansion of the pioneer forest in Trabaque Canyon had significant pulses. At the top of the Trabaque section, the proportion of *Pinus sylvestris/nigra* pollen increased (Supplementray Figs 7 and 8). This change suggests drier and/or cooler conditions, in agreement with equivalent changes in the composition of forests over Europe at that time[6,8].

References

1. Drysdale, R.N. *et al*. Evidence for obliquity forcing of glacial Termination II. *Science* **325**, 1527-1531 (2009).
2. Mokeddem, Z., McManus, J.F. & Oppo, D.W. Oceanographic dynamics and the end of the last interglacial in the subpolar North Atlantic. *Proc. Natl. Acad. Sci. U.S.A.* **111**, 11263-11268 (2014).
3. Fairchild, I.J. *et al*. Controls on trace element (Sr-Mg) compositions of carbonate cave waters: implications for speleothem climatic records. *Chem. Geol.* **166**, 255-269 (2000).
4. Domínguez-Villar, D., Vázquez-Navarro, J.A. & Krklec, K. The role of gypsum and/or dolomite dissolution in tufa precipitation: lessons from the hydrochemistry of a carbonate–sulphate karst system. *Earth Surf. Process. Landf.* **42**, 245–258 (2017).
5. Tzedakis, P.C., Frogley, M.R. & Heaton, T.H.E. Last Interglacial conditions in southern Europe: evidence from Ioannia, northwest Greece. *Glob. Planet. Change* **36**, 157-170 (2003).
6. Sánchez-Goñi, M.F. *et al*. Increasing vegetation and climate gradient in Western Europe over the last glacial inception (122-110 ka): data-model comparison. *Earth Planet. Sci. Lett.* **231**, 111-130 (2005).
7. Camuera, J. *et al*. Vegetation and climate changes during the last two glacial-interglacial cycles in the western Mediterranean: A new long pollen record from Padul (southern Iberian Peninsula). *Quat. Sci. Rev.* **205**, 86-105 (2019).
8. Brewer, S., Guiot, J., Sánchez-Goñi, M.F. & Klotz, S. The climate in Europe during the Eemian: a multi-method approach using pollen data. *Quat. Sci. Rev.* **27**, 2303-2315 (2008).

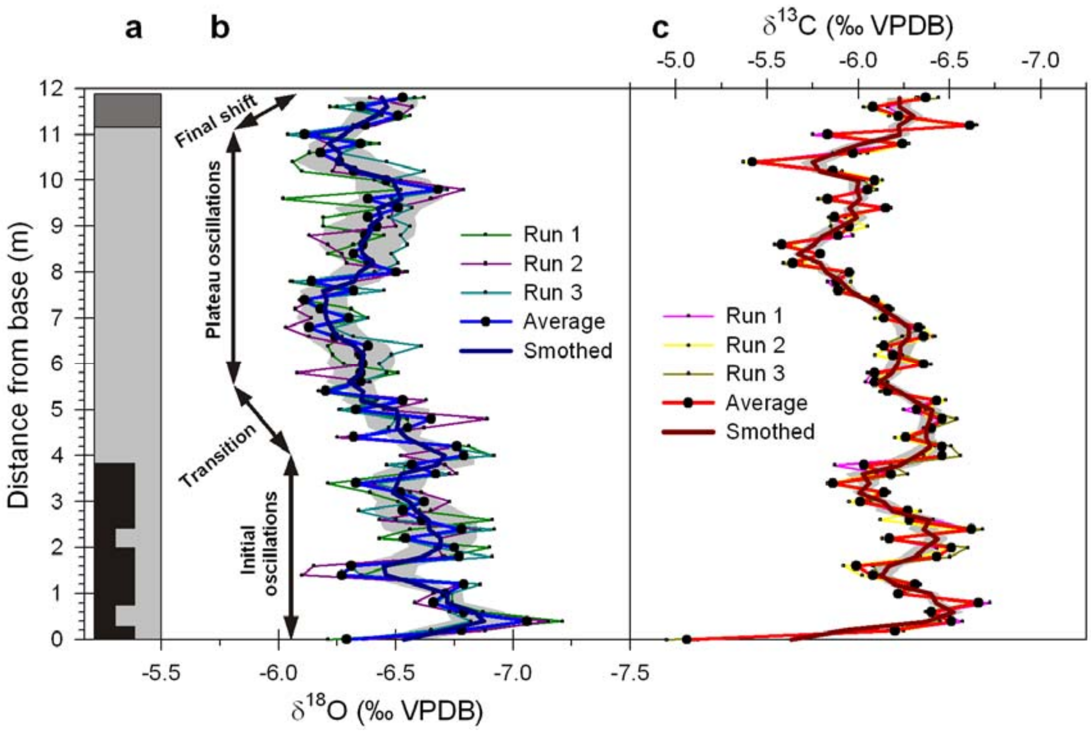

Figure S1. Trabaque tufa carbonate $\delta^{18}O$ and $\delta^{13}C$ records. (a) Simplified lithological log of Trabaque section. Patterns are for gravitational deposits (black) with distinct three pulses, well-cemented tufa sediments (clear grey), and alternation of well-cemented and loose tufa sediments (dark grey). (b) Tufa $\delta^{18}O$ record. The main modes of $\delta^{18}O$ variability are also illustrated. (c) Tufa $\delta^{13}C$ record. Three different samples from each depth were analyzed providing three different runs of the full section. The isotope values of the three runs were averaged and the grey shade shows the 1σ variability envelope. For a better visualization of the millennial oscillations 3-point running means smooth the isotope signals.

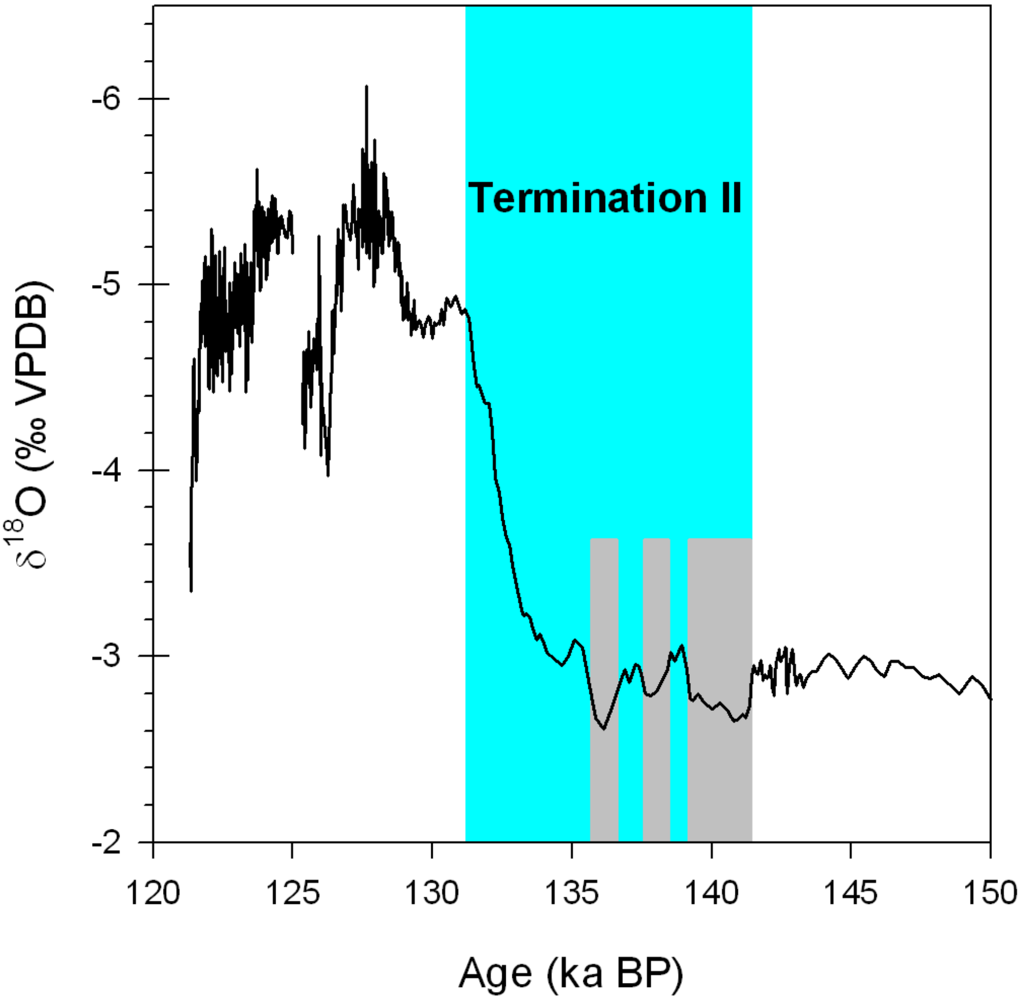

Figure S2. Millennial oscillation in the Corchia speleothem $\delta^{18}O$ record. Grey vertical bars indicate the dry periods of the millennial oscillation recorded during the first stage of the T-II. The duration of the T-II is shown as originally reported in the Corchia speleothem record[1].

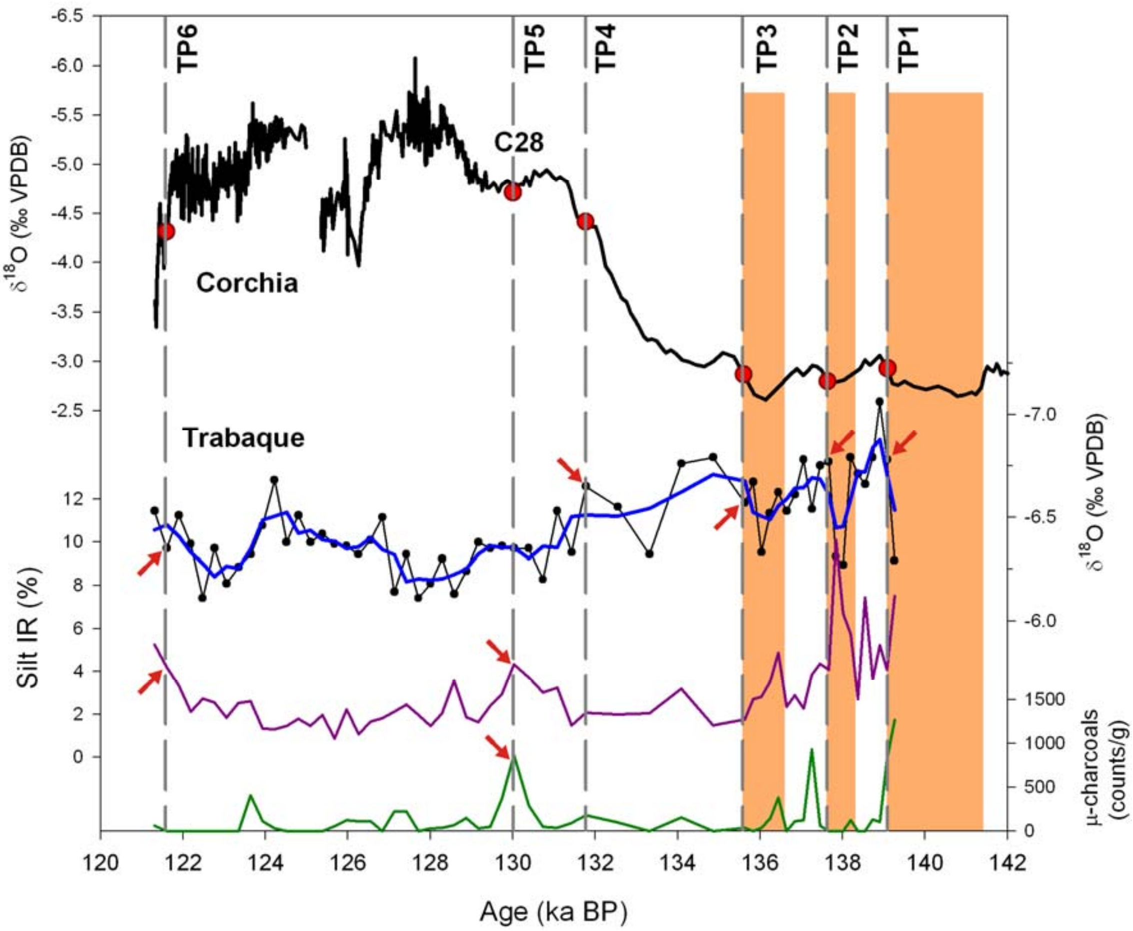

Figure S3. Tie points (TP) used for the synchronization of Trabaque record using Corchia age model[1]. The timing of every TP is illustrated by a vertical dashed line. The TP plotted on the Corchia speleothem $\delta^{18}O$ record are shown as red dots. TP on Trabaque $\delta^{18}O$ record or records from other proxies used for their identification are shown as red arrows. Vertical clear orange bars show the dry and erosive periods during the first stage of T-II. The timing of the oceanic cold event C28 is also shown. See the supplementary information text for detailed description of every TP.

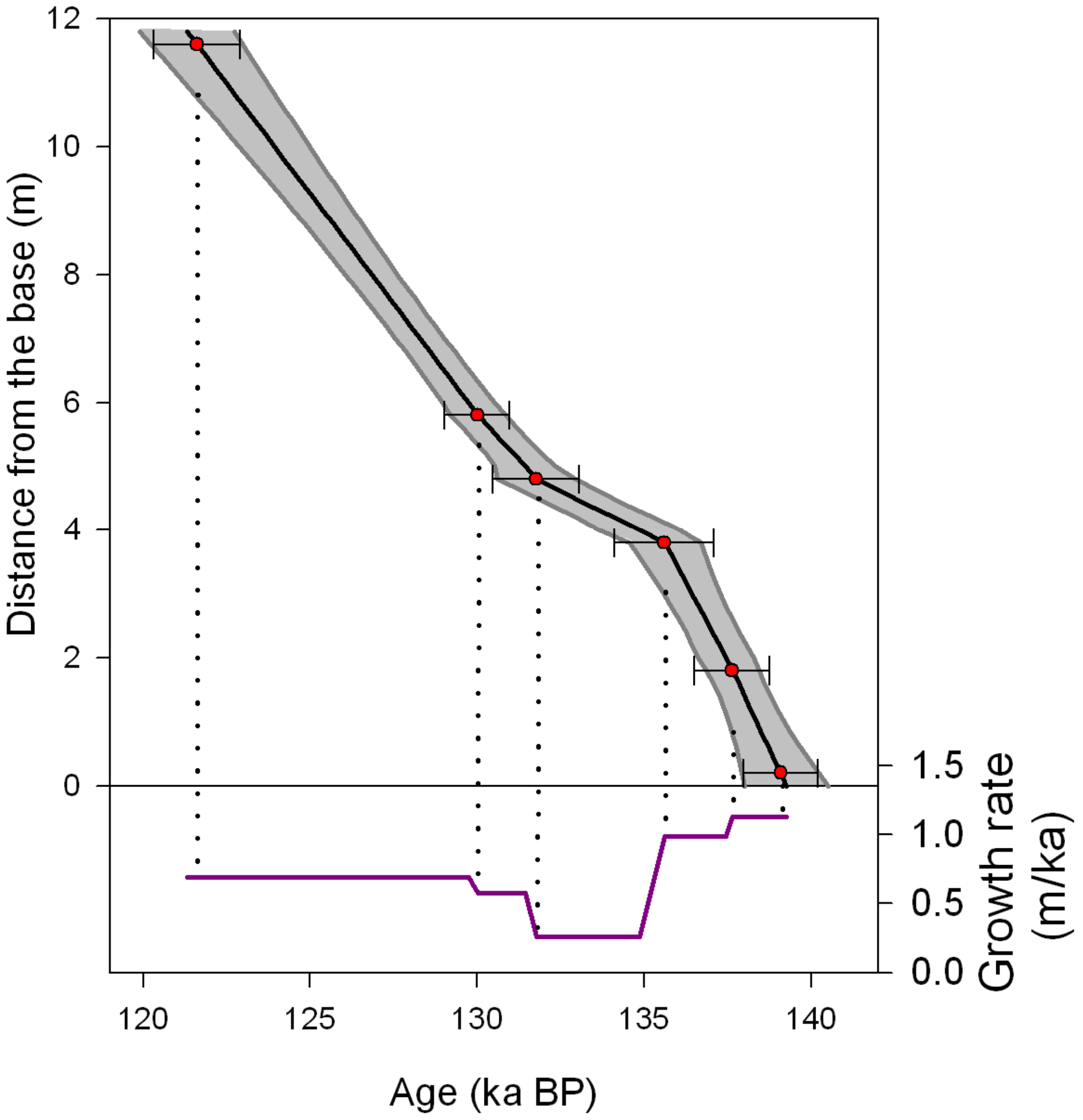

Figure S4. Trabaque record age model and growth rate. Selected TP and their uncertainties are plotted as red dots with error bars. The black line shows the age model of Trabaque record and the grey shade area is its 2σ uncertainty.

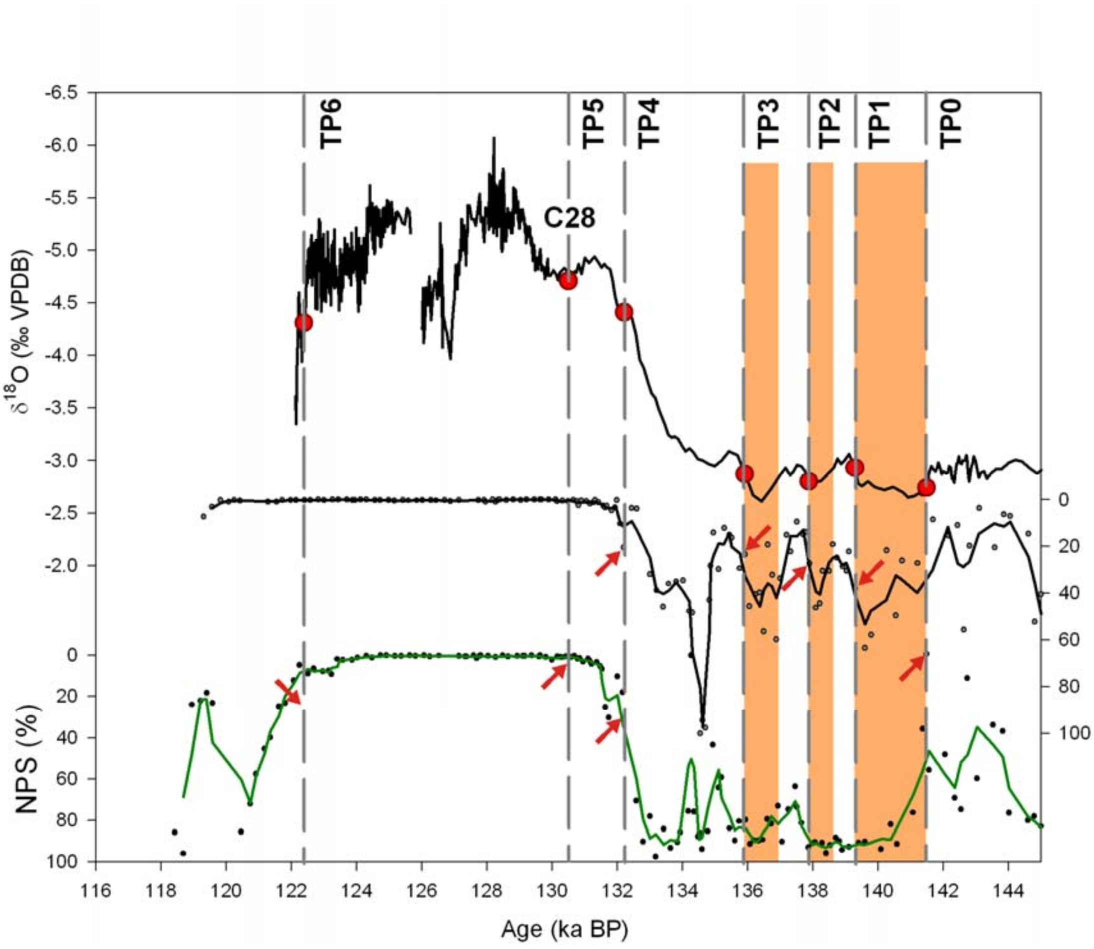

Figure S5. Tie points (TP) used for the synchronization of ODP 984 record[2] using Corchia age model[1]. The timing of every TP is illustrated by a vertical dashed line. The TP plotted on the Corchia speleothem $\delta^{18}O$ record are shown as red dots. TP on ODP 984 ice rafted debris (IRD) and *Neogloboquadrina pachyderma* sinistral cooling (NPS) percentage records used for their identification are shown as red arrows. Vertical clear orange bars show the dry and erosive periods during the first stage of T-II. The timing of the oceanic cold event C28 is also shown. See the supplementary information text for detailed description of every TP.

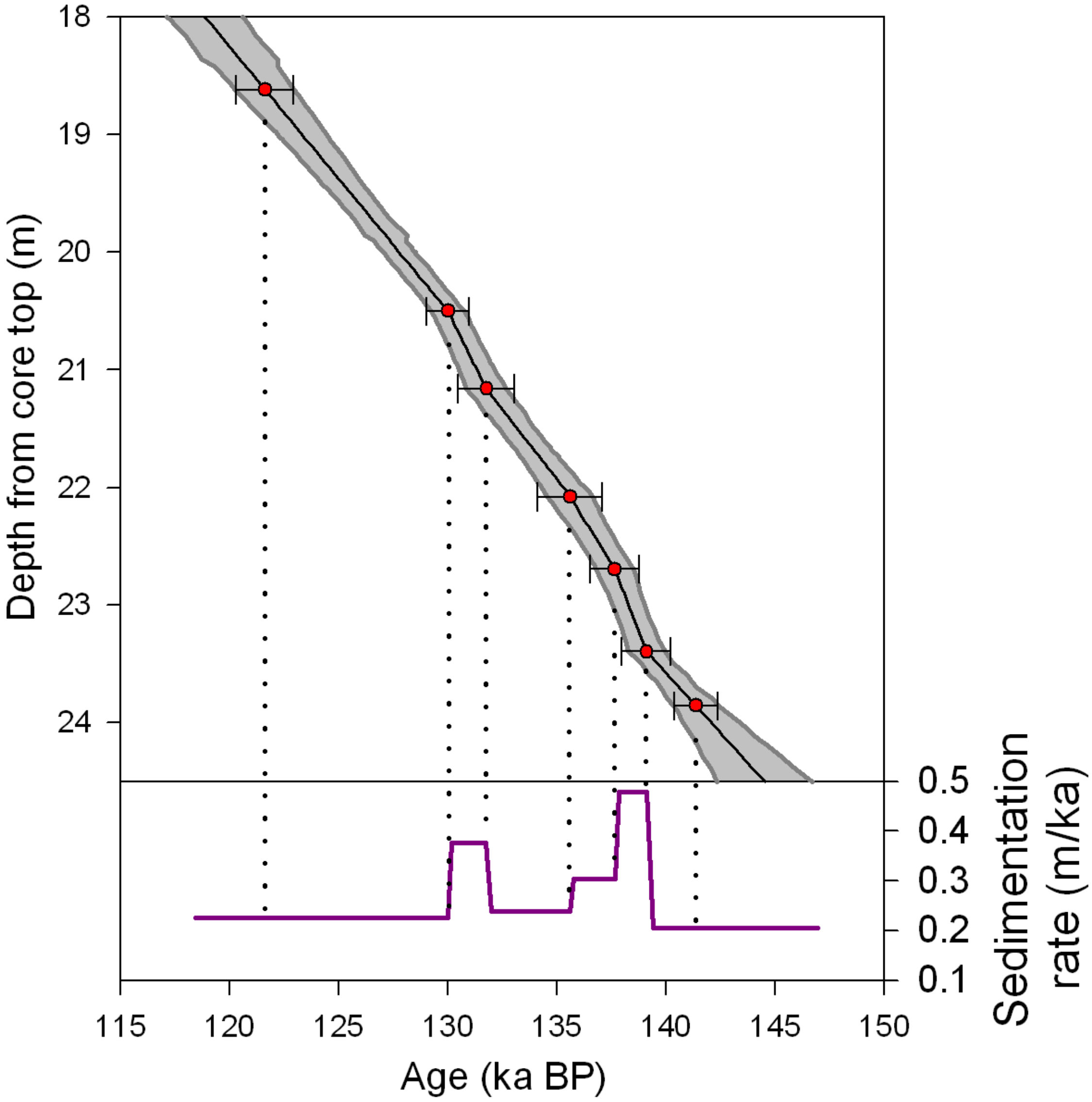

Figure S6. ODP 984 record age model and growth rate. Selected TP and their uncertainties are plotted as red dots with error bars. The black line shows the age model of the ODP 984 record and the grey shade area is its 2σ uncertainty.

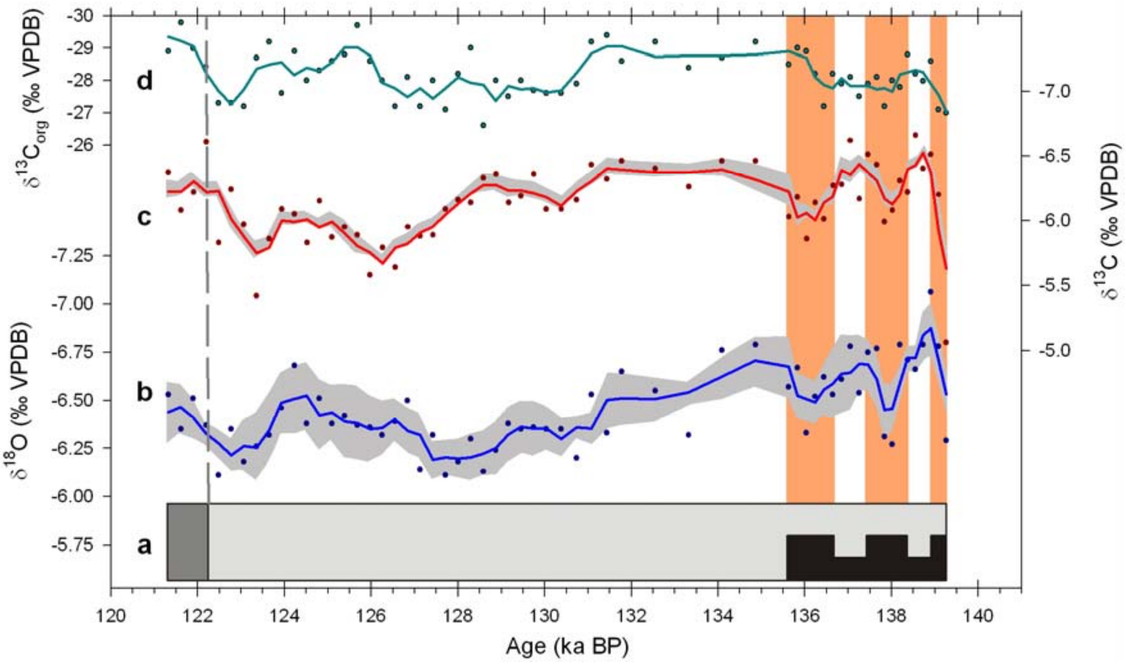

Figure S7. Multi-proxy record of Trabaque tufa deposit. (a) Synthetic log of Trabaque section. Patterns are for gravitational deposits (black) with distinct three pulses, well-cemented tufa sediments (clear grey), and alternation of well-cemented and loose tufa sediments (dark grey). (b) and (c) Tufa $\delta^{18}O$ and $\delta^{13}C$ records. Isotope values at each date (dots) are the average of 3 sub-samples and blue/red line is a 3-point running mean. The grey shade shows the 1σ variability of the three sub-samples along the record. (d) Record of carbon isotope composition of the organic matter contained in tufa ($\delta^{13}C_{org}$). Dots are raw data and the line is a 3-point running mean. This figure continues in the next page.

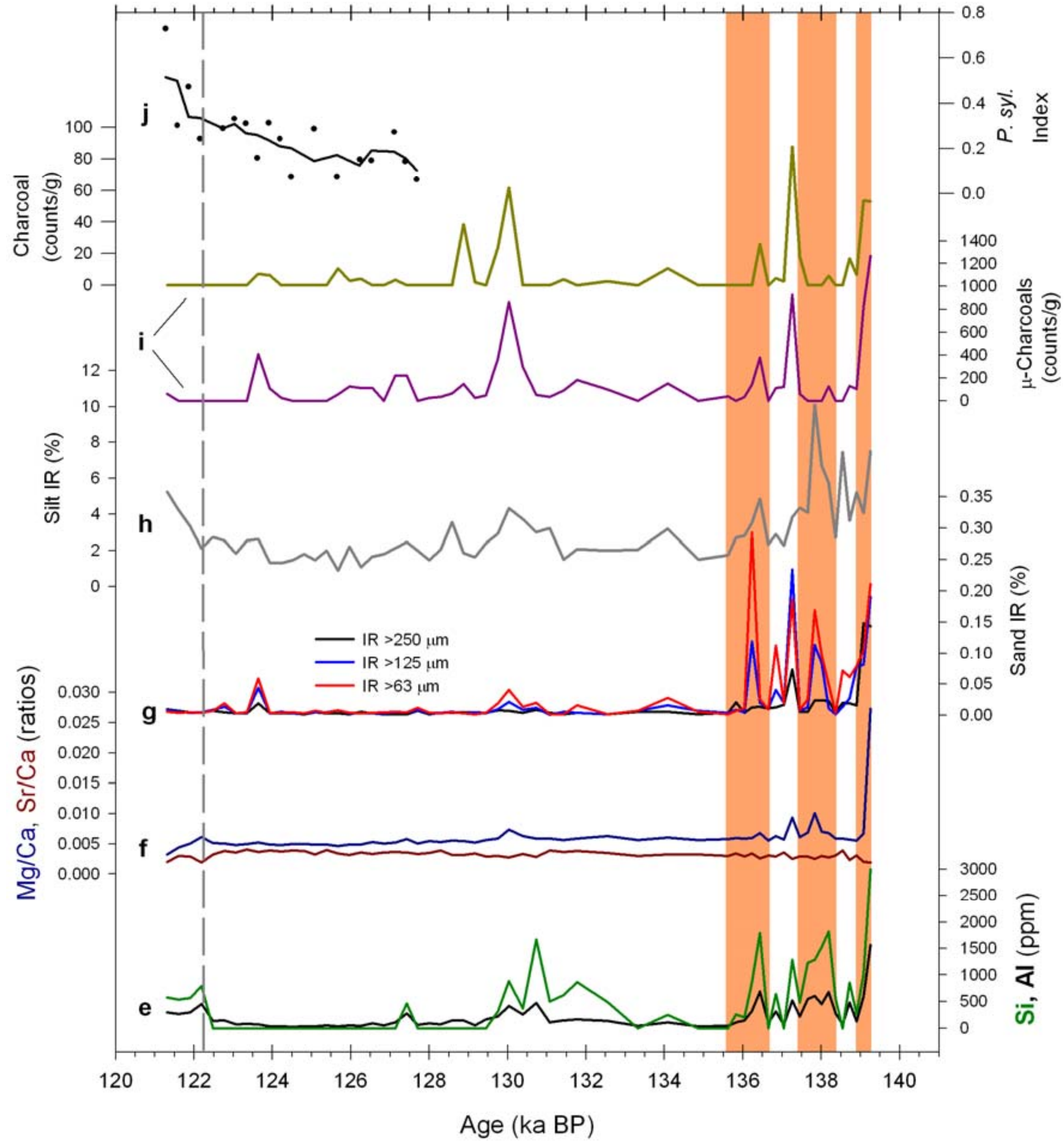

Figure S7 (continuation). Multi-proxy record of Trabaque tufa deposit. (e) Si and Al concentration on tufa samples. (f) Mg/Ca and Sr/Ca weight ratios in tufa carbonates. (g) Percentage of sand fractions 63-125 µm, 125-250 µm and >250 µm in the insoluble residue (IR) from well cemented tufa samples. (h) Percentage of silt IR fraction from well cemented tufa samples. (i) Abundance of micro-charcoal particles (50-125 µm) and charcoal particles (>125 µm). (j) *Pinus sylvestris* index is the ratio in pollen percentage between *P. sylvestris/nigra* and the rest of dominant non-riparian arboreal trees.

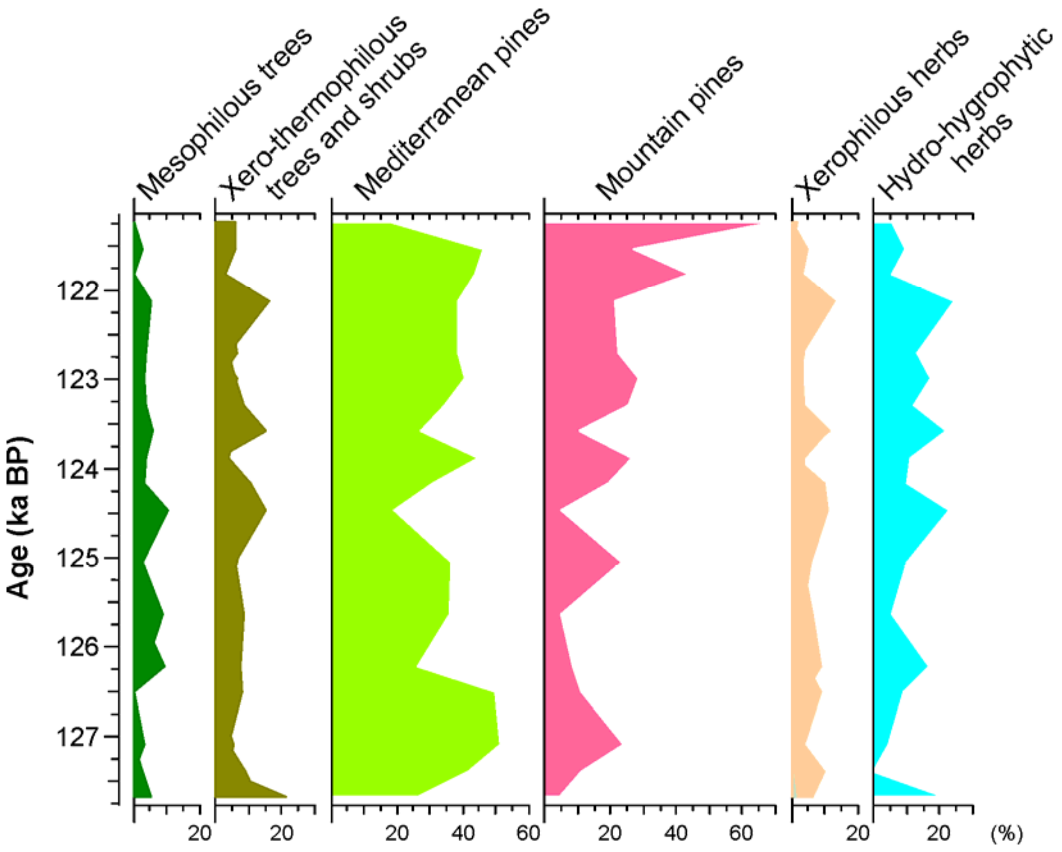

Figure S8. Synthetic pollen diagram from Trabaque record. Mesophilous trees are *Alnus*, *Betula*, *Celtis*, *Corylus*, *Fraxinus* and deciduous *Quercus*. Xero-thermophilous trees and shrubs are *Olea europea*, evergreen *Quercus*, *Juniperus*, *Cistus ladanifer* and Lamiaceae. Medierranean pines are represented by *Pinus pinaster*. Mountain pines are represented by *Pinus sylvestris/nigra*. Xerophilous herbs are Poaceae, Asteraceae, *Armeria/Limonium*, *Chenopodiaceae*, Brassicaceae, *Plantago coronopus* and Caryophyllaceae. Hydro-hygrophytic herbs are *Typha* and Cyperaceae.

Table S1. Tie points of age models for Trabaque and ODP 984 records.

TP	Corchia Cave speleothems		Trabaque tufa deposit		Ocean record ODP 984	
	Age (ka B2007)	Age (ka BP)	Depth* (m)	Age (ka BP)	Depth** (m)	ODP 984 (ka (BP)
0	141.423	141.366	-	-	23.86	141.367
1	139.151	139.094	0.2	139.080	23.40	139.111
2	137.690	137.633	1.8	137.659	22.70	137.649
3	135.659	135.602	3.8	135.627	22.08	135.608
4	131.822	131.765	4.8	131.776	21.16	131.746
5	130.060	130.003	5.8	130.033	20.50	129.994
6	121.665	121.608	11.6	121.607	18.62	121.624

*Distance from base

** Depth from core top
